# Missed opportunities to prevent HIV infections among pre‐exposure prophylaxis users: a population‐based mixed methods study, San Francisco, United States

**DOI:** 10.1002/jia2.25472

**Published:** 2020-04-15

**Authors:** Matthew A Spinelli, Nicole Laborde, Patrick Kinley, Ryan Whitacre, Hyman M Scott, Nicole Walker, Albert Y Liu, Monica Gandhi, Susan P Buchbinder

**Affiliations:** ^1^ University of California, San Francisco, Division of HIV, ID, and Global Medicine San Francisco CA USA; ^2^ San Francisco Department of Public Health San Francisco CA USA; ^3^ Graduate Institute of International and Development Studies Department of Sociology and Anthropology Geneve Switzerland

**Keywords:** PrEP, HIV seroconversion, Adherence, PrEP persistence, mixed methods, PrEP discontinuation

## Abstract

**Introduction:**

Pre‐exposure prophylaxis (PrEP) is highly effective, although PrEP adherence and persistence has been variable during real world implementation. Little is known about missed opportunities to enhance PrEP adherence among individuals who later HIV seroconverted after using PrEP. The goal of this analysis was to identify all HIV infections among individuals who had accessed PrEP in an integrated health system in San Francisco, and to identify potentially intervenable factors that could have prevented HIV infection through in‐depth interviews with people who HIV seroconverted after using PrEP.

**Methods:**

We identified individuals who initiated PrEP in an integrated safety‐net public health system and performed in‐depth chart review to determine person‐time on and after stopping PrEP over six years. We identified all PrEP seroconversions using the Centers for Disease Control and Prevention’s Enhanced HIV/AIDS Reporting System and then calculated HIV incidence while using PrEP and during gaps in use. We then performed in‐depth interviews with those who seroconverted.

**Results:**

Overall, 986 initiated PrEP across the San Francisco Department of Public Health from July 2012 to November 2018. Data were gathered from 895 person‐years on PrEP and 953 after stopping PrEP. The HIV incidence was 7.5‐fold higher after stopping PrEP compared to while on PrEP (95% CI 1 to 336). Of the eight individuals who HIV seroconverted; only one was taking PrEP at the time of seroconversion but was using on‐demand PrEP inconsistently. All eight agreed to qualitative interviews. Major barriers to PrEP persistence included substance use, mental health and housing loss; difficulty accessing PrEP due to cost, insurance, and the cost and time of medical visits; difficulty weighing PrEP’s benefit versus self‐perceived risk; and entering a primary partnership. The individual who developed HIV using on‐demand PrEP reported confusion about the dosing regimen and which sexual encounters required accompanying PrEP dosing.

**Conclusions:**

HIV incidence during gaps in PrEP use was nearly eight‐fold higher than while on PrEP in this large cohort in San Francisco. Many individuals who stop PrEP remain at risk of HIV, and participants reported that proactive outreach could potentially have prevented HIV infections. Individuals using non‐daily PrEP may require additional education and support in the United States.

## 1 Introduction

Pre‐exposure prophylaxis (PrEP) is a highly effective biomedical prevention strategy when taken during periods of HIV risk [1, 2]. Although PrEP persistence (sustained PrEP use over time) was high among PrEP users in clinical trials and early demonstration projects [2, 3, 4], it has been variable among later PrEP adopters and in real‐world evaluations, with 15 to 62% of users discontinuing PrEP by six months [5, 6, 7, 8, 9, 10, 11, 12, 13, 14]. Prevention‐effective adherence is a concept which describes the importance of using PrEP during periods of HIV risk, as compared to antiretroviral therapy, for which lifelong adherence is required [15]. Stopping PrEP during periods where individuals are not at risk of HIV may decrease toxicity, cost and side effects [15]. However, it is important to understand the reasons for stopping PrEP among individuals who have ongoing HIV risk and whether stopping PrEP is associated with a subsequent increase in the rate of new infections.

Despite many individuals stopping PrEP due to low self‐perception of risk, prior studies have also shown relatively large numbers of HIV infections after PrEP discontinuation [10, 11, 12]. In a prior analysis, we found that African‐Americans, transgender women and younger participants were more likely to discontinue PrEP in our system, in spite of disproportionate HIV incidence in these groups [16]. In this study, we first sought to understand to what extent HIV risk persists during PrEP gaps, via the HIV incidence rate, when compared to periods of active PrEP use in a large cohort. For this aim, we performed in‐depth chart review to categorize periods on and off PrEP among San Francisco Department of Public Health (SFDPH) primary care PrEP users, and used the SFDPH/Centers for Disease Control and Prevention (CDC) enhanced HIV surveillance system to rigorously ascertain HIV diagnoses [17]. We then sought to understand PrEP use patterns by performing in‐depth interviews with individuals who had HIV seroconverted after or while using PrEP with the goal of understanding the circumstances which led them to stop PrEP or use it inconsistently. The goal of these analyses was to understand potential missed opportunities for intervention to support PrEP persistence among individuals with ongoing exposure to HIV.

## 2 Methods

### 2.1 Population and outcome ascertainment

Data for individuals prescribed PrEP in the San Francisco Primary Care Clinics (SFPCC), a 14‐clinic integrated, safety‐net municipal health system, were analysed 7/2012‐11/2018. There are approximately 60,000 clients within the health system. Clients are predominantly publicly‐insured, with uninsured clients (overall 9%) supported by a municipal access programme, with PrEP available free of charge through access programmes. PrEP 2‐1‐1/on‐demand was not routinely offered within the SFPCC at the time of this analysis. Participants were classified as being on or off PrEP based on a combination of documentation in the electronic medical record (EMR) and via data obtained through direct outreach from the SFDPH PrEP coordinators, who attempted to contact PrEP users lost to follow‐up and record use [16]. The SFPCC is an integrated health system, and medication reconciliation is performed and documented in the EMR at every visit, limiting unrecorded PrEP use outside the system. For the 7% of PrEP users for whom no information on a stop date could be obtained from the EMR or via direct outreach, we calculated the last date a prescription would have been available according to EMR prescription, assuming daily dosing. Person‐time was censored during periods in which participants were disenrolled. Discontinuations/gaps were defined as 90 days without PrEP [9]. HIV seroconversions were ascertained using the SFDPH/CDC’s enhanced HIV surveillance system (eHARS), permitting collection of HIV diagnoses that occurred both within and outside of the SFDPH’s surveillance jurisdiction through mandated HIV diagnosis reporting. HIV diagnoses were classified as occurring while using PrEP or after stopping PrEP/during a gap in use based on timing of the HIV diagnosis and PrEP start/stop date for each individual. PrEP start and stop dates were also used to calculate person‐time (1) on PrEP and (2) during gaps in use, through November 2018. Confidence intervals for HIV incidence were calculated using Poisson models, and HIV incidence off PrEP was compared to HIV incidence while on PrEP.

### 2.2 Interviews and qualitative analysis

All individuals who developed HIV infection after having initiated PrEP between July 2012 and November 2018 were invited for in‐depth interviews. Interviews were approximately one hour in length and occurred in person or over the phone based on participant preference. Interviews were performed by one of two medical anthropologists or an infectious diseases physician, audio‐recorded and transcribed. A code book was first developed using the Motivational PrEP Cascade and the situated Information‐Motivation‐Behavioural Skills theoretical models [18, 19, 20] and was subsequently iteratively revised based on emergent themes [21]. One‐tenth of transcripts were double‐coded, and the research team discussed discrepancies and revision of the code book accordingly. At the end of interviews, the team defined 37 analytic codes. Once coding was completed using Dedoose analytic software, the team ran code reports for 18 of these codes, creating brief summaries of each excerpt. Themes were summarized with a view towards identifying overarching themes relevant to PrEP use and PrEP discontinuation/gaps in use in individuals who subsequently experienced PrEP failure [22] and to identify missed opportunities for health system or provider intervention. This study was approved by the University of California, SF Institutional Review Board. All interview participants provided informed consent and the study was conducted in accordance with the Declaration of Helsinki.

## 3 Results

### 3.1 Population of SFDPH primary care clinics PrEP users

Overall, 986 individuals initiated PrEP across the SFDPH primary care clinics 7/2012‐11/2018. Two‐thirds (66%) were men who have sex with men (MSM), 14% were transgender women who have sex with men, 19% were heterosexuals and 1% were people who inject drugs. The median age was 35 years: 7% were Asian, 14% Black, 27% Latinx, 37% White and 15% mixed/other (Table1). The median time on PrEP was eight months. There were 953 person‐years of observation while on PrEP and 895 person‐years of observation after stopping PrEP/during a gap in use. Seven percent of the sample stopped and later restarted PrEP.

**Table 1:** Characteristics of San Francisco Primary Care Clinics (SFPCC) PrEP users

	Overall	Experienced seroconversion on PrEP
	N (%)	N (%)
Total	986 (100)	8 (100)
Age		
<25	177 (18)	3 (38)
25 to 39	454 (46)	2 (25)
40 to 64	345 (35)	3 (38)
65+	10 (1)	0 (0)
Female Birth Sex	148 (15)	0 (0)
Male Birth Sex	838 (85)	8 (100)
Race/ethnicity		
African‐American	138 (14)	2 (25)
Asian	69 (7)	0 (0)
Latinx	266 (27)	2 (25)
White	365 (37)	3 (38)
Mixed/Other	148 (15)	1 (13)
PrEP indication		
Heterosexual	187 (19)	0 (0)
MSM	651 (66)	5 (63)
PWID	10 (1)	0 (0)
TGWSM	138 (14)	3 (38)
PrEP initiation year		
2012 to 14	66 (7)	1 (13)
2015 to 16	278 (28)	3 (38)
2017 to 18	642 (65)	4 (50)

MSM, men who have sex with men; PrEP, pre‐exposure prophylaxis; PWID, people who inject drugs; TGWSM, transgender women who have sex with men.

### 3.2 HIV Incidence on and after Stopping PrEP

Overall, eight HIV infections were identified, with one occurring outside of the SFDPH jurisdiction. Of the eight HIV infections, one occurred while using PrEP, leading to an HIV incidence of 0.1 per 100 person‐years during PrEP use (95% Confidence Interval (CI): 0.003 to 0.5). There were seven HIV infections after during gaps in use, leading to an HIV incidence of 0.8 per 100 person‐years during this period (95% CI: 0.3 to 1.6). The HIV incidence rate was 7.5‐fold higher in off‐PrEP periods compared to the rate during periods on PrEP (95% CI: 1.0 to 336).

### 3.3 In‐depth interview themes

Of the eight individuals contacted who HIV seroconverted during or after using PrEP, all agreed to participate in in‐depth interviews. Overall, two were Black, two were Latinx, one was Native American and three were White. Three were younger than age 25, and two were age 50 or older; five were MSM and three were transgender women (Table1). Of the seven participants who had discontinued PrEP, four did not discuss the decision to stop PrEP with their clinician, and only one reported intending to stop using PrEP indefinitely. Two participants engaged in sex work, four reported methamphetamine use, and three reported unstable housing. The major themes from the interviews included: (1) difficulty accessing PrEP due to substance use, mental health or housing challenges (seven participants); (2) difficulty prioritizing PrEP given the effort or cost required to remain on PrEP (five participants); (3) difficulty assessing one’s potential benefit from PrEP versus the risk of HIV (five participants); and (4) desire for trust and intimacy as a reason to stop PrEP (two participants).

### 3.4 Difficulty using PrEP due to substance use, mental health or housing challenges

Of the seven participants who discontinued PrEP, all seven identified a major substance use, mental health, or housing challenge that contributed to them stopping PrEP. Four of these participants described substance use as having a marked impact on their ability to take PrEP. Participants described difficulty tracking appointment times, lab monitoring visits, or adherence. In particular, the use of methamphetamine and other drugs during sex, that is, chemsex, had an impact on their PrEP adherence and continued use: “P and P. They call it play and party. I think it's a big connection right there [with adhering to PrEP around times of sexual encounters]” (Participant (P) 1). Another participant discussed blacking out due to alcohol and not realizing he had condomless sex, leading him to underestimate his risk of HIV when deciding whether or not to restart PrEP (P2). One participant felt that undertreated depression, and its intersection with his substance use, was a major factor. He recommended additional training for PrEP providers in mental health issues:

“you may probably be aware that especially the providers, [need] to be given more tools and training…in prevention and mental illness because, for me, I'm pretty sure it's one of the biggest things” (P3).

Finally, the two participants who lost housing while using PrEP identified it as a key factor in their PrEP discontinuation: “Well, so I lost my housing, and so in the midst of all that, there's just no going to doctor appointments” (P4). The other participant reported not being able to access pill bottles or identity documents after being kicked out of his housing arrangement suddenly.

### 3.5 Difficulty prioritizing PrEP given the effort/cost required to remain on PrEP

Five of the participants reported that accessing PrEP required significant effort to maintain active insurance, navigate PrEP access programmes, and attend visits with a PrEP provider. These efforts and costs were weighed against difficulty keeping up with busy work and school schedules and addressing substance use, mental health or housing concerns. Quarterly labs and provider visits, receiving only 30‐day prescriptions, and difficulty contacting their provider or scheduling appointments contributed to gaps in their PrEP use. Change in employment resulting in loss or change in insurance, or the need for travel, were particularly vulnerable times: “After I moved I really didn’t know how to find a new doctor so I let it slip” (P5). Participants discussed the perceived cost and complicated process to obtain insurance as a significant barrier:

“No. I mean, maybe if they did tell me I should apply for Medi‐Cal [public insurance] or something…that probably would've been easier. Because as soon as I saw my $300 copay, well, that's not going to happen, so I’m done” (P4).

For the four PrEP users who did not discuss discontinuation with their provider, all agreed that proactive outreach from clinical staff, ideally through a text message or call, would have been appreciated.

### 3.6 Difficulty weighing PrEP’s benefit versus the risk of HIV

Participants reported having greater motivation to stay on PrEP during periods when they had greater number of partners. However, during periods when they were off PrEP due to entering a stable partnership or having fewer partners, five participants reported less motivation to maintain a PrEP prescription given the effort required, “when I’m going out less, it definitely impacts how fast I work to get more [PrEP]” (P2). One participant, who engaged in sex work and intermittently used condoms, stopped PrEP due to side effects, but thought she would have been able to manage the side effects if her providers had been direct about her potential HIV risk:

“If I had someone stop me and tell me, hey, this is really serious like, you're going to contract it, if you don't take the PrEP … I wish I would have just monitored myself better, or had someone else monitoring me better because I was like a risk factor” (P5).

However, some participants also reported that emphasis on needing to use PrEP because of “risk behaviour” could be stigmatizing. One participant in particular felt motivated to try to change his sexual behaviour rather than continuing to take PrEP. He then later regretted stopping PrEP:

“I talked to my doctor and we both decide to get off PrEP because it didn’t work that well for me [due to concerns about how he was perceived by his community]. But my behaviour didn't stop right there” (P6).

The individual who acquired HIV while using PrEP had previously switched from daily PrEP to on‐demand dosing on the recommendation of a provider given his worsening chronic kidney disease. He reported confusion about taking one versus two tablets prior to sex and which types of sexual encounters required on‐demand PrEP. He requested additional training for providers in educating patients about the use of on‐demand PrEP and the “risks and benefits of switching to on‐demand dosing” (P7).

### 3.7 Desire for trust and intimacy within a relationship counters motivation to remain on PrEP

For two participants, the desire for intimacy and trust countered motivation to remain on PrEP. They reported entering a primary partnership and felt that continuing PrEP would be interpreted as distrust in their partner, “I'm betraying a trust if I feel I need to protect myself (P5)”. Entering a primary partnership did not necessarily mean that they stopped seeing outside partners or that the relationship was mutually monogamous, making these participants vulnerable to HIV infection. Another reported her partner’s discomfort with PrEP use because he believed it implied she had outside partners, “Like, that is like what it's for basically, is so people can have guilt‐free unprotected sex (P8).” A desire to develop a primary partnership further and show trust in their partners conflicted with conceptions of PrEP as a “drug for people with multiple partners.”

## 4 Discussion

Among nearly 1000 PrEP users in a United States‐based Department of Public Health municipal primary care delivery system, HIV incidence was nearly eight‐fold higher after stopping PrEP than during periods when individuals were taking PrEP. Among the seven individuals who stopped PrEP and subsequently developed HIV, factors impacting continued PrEP use included substance use, mental health and housing concerns; difficulty accessing PrEP due to cost, insurance and the need for medical and laboratory visits; difficulty weighing PrEP’s benefit versus one’s self‐perceived HIV risk; and entering a primary partnership. HIV incidence after stopping PrEP was similar to that in a Boston LGBT primary care clinic [11 but lower than that seen in a Los Angeles LGBT primary care clinic [10 and Montreal sexual health clinic [12] potentially due to our study being performed among a general primary care population. A strength of this analysis is that we were able to rigorously ascertain HIV diagnoses by leveraging the SFDPH/CDC’s enhanced HIV surveillance system.

Our study highlights the importance of social determinants of health, such as access to housing, mental health care, and substance use treatment, for people using PrEP. Among U.S. people living with HIV (PLHIV), stable housing status is associated with achieving viral suppression [23] receipt of preventive health care [24 and lower mortality [25]. Given that two of the seven individuals who developed HIV infection after stopping PrEP did so in part because of losing their housing, there is an urgent need to address the U.S. HIV risk/infection and homelessness syndemic [26]. Furthermore, the intersection of substance use and difficulties accessing and adhering to prevention strategies among participants highlights the need for integrated care strategies. Additional resources for treatment of substance use disorder are needed within PrEP clinics and primary care settings, such as counselling, social workers, providers who can prescribe buprenorphine for opioid use disorder, harm reduction education, referral to syringe exchange programmes, and naloxone distribution. Although novel interventions for stimulant use disorder are needed, it should be noted that with sufficient support, stimulant users can achieve high PrEP adherence [27, 28, 29]. One participant specifically requested that mental health services be co‐located within primary care clinics that offer PrEP [30]. Integrated solutions which address housing instability, substance use and mental health challenges will be therefore be needed to ensure disparities in HIV infection do not worsen among vulnerable populations, with novel care models currently in development [31, 32].

Actual and perceived costs, and difficulty maintaining active insurance coverage, were major barriers in this population to ongoing PrEP use. Despite initially accessing PrEP successfully, most of the participants reported challenges maintaining their access to PrEP. Competitive pricing of authorized generic PrEP could alleviate these barriers. Although co‐pay assistance programmes are available, many PrEP users are not aware of their availability, and the procedures required to obtain PrEP coverage are significant barriers in the United States compared to other countries where PrEP is available through national health systems. Insurance churn among participants, who cycled on and off public insurance when their employment status changed, also contributed to gaps in PrEP use in this cohort. It is clear that simplified coverage procedures and decreased cost of PrEP in the United States and elsewhere is urgently needed to fulfil PrEP’s prevention potential [33, 34].

Even when co‐pay assistance is available, the time, transportation needs and costs of care required for ongoing PrEP prescriptions is burdensome for many PrEP users. Pharmacy‐/ telehealth‐delivered PrEP, and express visit systems, in which an initial triage is performed on computer tablets to allow stable PrEP users to defer in‐person medical provider visits, are potential solutions that deserve additional study [35, 36, 37]. The recently‐passed California bill‐159, which allows pharmacists to dispense 60 days of PrEP without prescription, is likely to support uptake [37]. However, alternative delivery systems will also need to provide support for PrEP users who are struggling with adherence or could benefit from additional services such as substance use treatment. For instance, mobile health strategies such as asynchronous two‐way messaging could allow large PrEP programmes to efficiently target support to individuals who request help [8]. Even within a traditional delivery setting, over half of those interviewed who stopped PrEP did not discuss it with their provider. Proactive outreach was highly acceptable and could have potentially triggered a discussion about barriers to PrEP use amenable to intervention, such as offering 90‐day PrEP prescriptions, providing benefits navigation or arranging follow‐up via telephone visits when participants cannot attend in‐person [38]. Proactive outreach/navigation, including when performed by non‐clinicians, has been shown to improve retention in care for PLHIV and support PrEP uptake [39, 40, 41].

Participants reported difficulties assessing whether they remained at sufficient risk of HIV to justify the effort required to continue PrEP. Participants viewed PrEP as a prevention solution for people with multiple partners. Stigma reinforced by the association of PrEP with risky behaviour contributed to the decision of several of the participants to stop PrEP. Unfortunately, for many populations, HIV risk is driven not by behaviour but by one’s sexual network and/or one’s partners’ sexual network [42, 43]. For this reason and others, the CDC guidelines predict HIV risk poorly among key populations such as black MSM and heterosexual women [42, 43]. In addition to removing barriers to PrEP delivery, PrEP should be normalized as a routine preventive health procedure for people potentially exposed to HIV. This approach could spur uptake and persistence, while avoiding reinforcement of PrEP stigma. Finally, additional tools and frameworks are needed to guide patient and provider decision‐making around PrEP discontinuation.

Lastly, on‐demand or 2‐1‐1 PrEP is an attractive strategy for people who may not want to take a daily pill for HIV prevention [2]. However, there is currently limited use of 2‐1‐1 PrEP in the United States, although the International Antiretroviral Society‐U.S.A guidelines endorse this strategy, and there is high interest among MSM and transgender women not using daily PrEP [44, 45]. Educational resources for 2‐1‐1 PrEP should be made more widely available, and they should emphasize the importance of taking 2‐1‐1 PrEP with all sexual encounters. Additional tools, to support decision‐making to initiate 2‐1‐1 PrEP, and dosing reminders for 2‐1‐1, deserve additional study.

There were several limitations of our study. We cannot exclude inaccuracies in self‐reported PrEP use. For the 7% of participants for whom a stop date was not available, leading us to rely on prescription data, it is possible that calculated stop dates underestimate if participants used PrEP in a non‐daily fashion. We are unable to assess whether people who stopped PrEP would have gone on to restart PrEP or if reported barriers could be impacted by recall/social desirability bias. Although almost a thousand participants were included in this cohort on PrEP, HIV seroconversion events were rare, decreasing precision of HIV incidence. Results may also not be generalizable outside of a U.S. primary care setting and/or to other health systems with differing demographics and HIV epidemic patterns.

## 5 Conclusions

HIV incidence was high among individuals with gaps in PrEP use in a general primary care setting. Integrated strategies to support PrEP users with substance use or mental health treatment, simplification of medication coverage while streamlining PrEP delivery, and proactive outreach to PrEP users can support PrEP persistence among individuals at ongoing risk of HIV. By virtue of previously requesting or being offered PrEP, former PrEP users are a key population for prevention interventions, either to support PrEP reinitiation or to offer alternative prevention strategies. As more and more individuals try PrEP in high HIV incidence areas supporting continued PrEP use during periods of risk will increasingly become a critical gap in the HIV prevention cascade.

## Competing Interests

The authors have no conflicts of interest to report.

## Authors’ Contributions

N.L., R.W. and M.A.S. performed the interviews. N.L., P.K., M.A.S., N.W. and H.M.S. performed the qualitative data analysis. M.A.S. performed the quantitative data analysis and wrote the paper. H.M.S., M.G., A.Y.L. and S.P.B. created the PrEP cohort. All authors revised the manuscript for content and have read and approved the final manuscript.
